# Dynamics of metabolic responses to periods of combined heat and drought in *Arabidopsis thaliana* under ambient and elevated atmospheric CO_2_

**DOI:** 10.1093/jxb/ery055

**Published:** 2018-02-15

**Authors:** Gaurav Zinta, Hamada AbdElgawad, Darin Peshev, James T Weedon, Wim Van den Ende, Ivan Nijs, Ivan A Janssens, Gerrit T S Beemster, Han Asard

**Affiliations:** 1 Centre of excellence PLECO (Plants and Ecosystems), Department of Biology, University of Antwerp, Universiteitsplein, Antwerp, Wilrijk, Belgium; 2 Integrated Molecular Plant Physiology Research (IMPRES), Department of Biology, University of Antwerp, Groenenborgerlaan, Antwerp, Belgium; 3 Department of Botany, Faculty of Science, Beni-Suef University, Beni-Suef, Egypt; 4 Laboratory of Molecular Plant Biology, Institute of Botany and Microbiology, KU Leuven, Leuven-Heverlee, Belgium

**Keywords:** Amino acids, carbohydrates, climate change, extreme events, gene expression, lipids, membrane composition, primary metabolism

## Abstract

As a consequence of global change processes, plants will increasingly be challenged by extreme climatic events, against a background of elevated atmospheric CO_2_. We analysed responses of *Arabidopsis thaliana* to periods of a combination of elevated heat and water deficit at ambient and elevated CO_2_ in order to gain mechanistic insights regarding changes in primary metabolism. Metabolic changes induced by extremes of climate are dynamic and specific to different classes of molecules. Concentrations of soluble sugars and amino acids increased transiently after short (4-d) exposure to heat and drought, and readjusted to control levels under prolonged (8-d) stress. In contrast, fatty acids showed persistent changes during the stress period. Elevated CO_2_ reduced the impact of stress on sugar and amino acid metabolism, but not on fatty acids. Integrating metabolite data with transcriptome results revealed that some of the metabolic changes were regulated at the transcriptional level. Multivariate analyses grouped metabolites on the basis of stress exposure time, indicating specificity in metabolic responses to short and prolonged stress. Taken together, the results indicate that dynamic metabolic reprograming plays an important role in plant acclimation to climatic extremes. The extent of such metabolic adjustments is less under high CO_2_, further pointing towards the role of high CO_2_ in stress mitigation.

## Teaser

Metabolic changes in Arabidopsis induced by periods of elevated heat and drought stress under ambient and elevated CO_2_, are dynamic and specific to different classes of molecules.

## Introduction

Extreme heat and drought often co-occur and dramatically reduce plant growth. They are predicted to become more frequent and severe as a consequence of global climate change (IPCC, 2012). Simultaneously, the predicted future atmosphere will contain higher CO_2_ levels, impacting plant growth and development. Heat, water deficit, and CO_2_ effects on plants are relatively well studied, although most often as separate factors (Ainsworth and Long, 2005; Dieleman *et al.*, 2012; Feng *et al.*, 2014; Pandey *et al.*, 2015). Considerably less is known about combined heat extremes and drought stress effects under elevated CO_2_, a scenario very relevant to global climate change.

Plant responses to heat and drought depend on the intensity and frequency of these events, and on plant-specific factors such as developmental stage and adaptation potential. Inhibition of photosynthesis, changes in cell metabolism, and deterioration of membranes and proteins are frequently observed under severe stress. Metabolic changes may lead to imbalances in redox homeostasis and elevated levels of reactive oxygen species (ROS), causing oxidative stress (Mittler, 2002; Foyer and Noctor, 2005; Krasensky and Jonak, 2012; Munné-Bosch *et al.*, 2013). Defences against short-term exposure to extreme heat or drought include regulation of stomatal opening and induction of protective molecules (e.g. osmolytes, heat shock proteins, and antioxidants) (Wang *et al.*, 2003; Vinocur and Altman, 2005; Wahid *et al.*, 2007; Ashraf, 2010).

Elevated atmospheric CO_2_ stimulates biomass production, particularly in plants with C_3_-carbon metabolism (fertilization effect) (Drake *et al.*, 1997; Long *et al.*, 2004; Ainsworth and Long, 2005; Pandey *et al.*, 2015). In addition, high CO_2_ stimulates respiration and alters flowering time (Springer and Ward, 2007; Leakey *et al.*, 2009). Elevated CO_2_ also reduces the impact of abiotic stresses on plants, such as ozone, heat, and drought (AbdElgawad *et al.*, 2016). The stress-mitigating effect on drought responses is in part caused by stomatal factors such increased stomatal closure and reduced stomatal density, which improve plant water-use efficiency (Ghannoum *et al.*, 2003). However, non-stomatal factors, including changes in photosynthetic enzymes, reduction of photorespiration (Aranjuelo *et al.*, 2008; Mishra *et al.*, 2013; Zinta *et al.*, 2014; AbdElgawad *et al.*, 2015), and increased levels of defence molecules (e.g. proline, antioxidants) are also important (Geissler *et al.*, 2010; Pintó Marijuan *et al.*, 2013; Li *et al.*, 2015).

To gain a mechanistic understanding of heat and drought effects under a predicted future climate, it is necessary to not only focus on a selected set of defence parameters, but also to obtain a broader view of metabolic changes. Determining system-wide changes in gene expression level has become relatively affordable, and studies that address transcriptome-level effects of abiotic stress are becoming increasingly common (Rogers *et al.*, 2006; Osuna *et al.*, 2007; Usadel *et al.*, 2008; Kanani *et al.*, 2010; Sulpice *et al.*, 2013). However, transcriptome changes do not result in linear alterations in protein activity and metabolite changes (Stitt and Gibon, 2014). Therefore, additional determination of changes in metabolite and enzyme activities provides a more conclusive view of the physiological reprogramming of the plant.

Analyses of metabolic changes have been performed for some abiotic stresses (Kaplan *et al.*, 2004; Rizhsky *et al.*, 2004; Sanchez *et al.*, 2008; Usadel *et al.*, 2008; Cramer *et al.*, 2011). These studies have revealed that plants respond to stresses by transient, sustained, early- and late-metabolic adjustments. For example, raffinose and proline accumulate to high levels over the course of several days of salt, drought, or cold treatment, whereas carbohydrate metabolism changes rapidly in a complex, time-dependent manner (Krasensky and Jonak, 2012). Moreover, some metabolic changes are common among stresses, whereas others are more stress-specific. For example, proline accumulates upon drought, salt, and low-temperature treatments, but not upon high-temperature stress (Krasensky and Jonak, 2012). Such responses highlight the complexity of metabolic adjustments in natural environments. However, there is little or no information on the metabolic alterations induced by climatic extremes (e.g. heat and drought) under current and predicted future climate CO_2_ levels.

In previous work, we analysed the effects of climate extremes (periods of elevated heat combined with drought) at the level of growth, photosynthesis, and oxidative stress responses (ROS, antioxidants) under ambient and elevated CO_2_ (Zinta *et al.*, 2014). Based on transcriptome and enzyme activity data, we concluded that the stress-mitigating CO_2_ effect is mediated by increased antioxidant capacity and reduced photorespiration. However, the transcriptome data also suggested significant changes in primary metabolism (Zinta *et al.*, 2014). Given the importance of elevated CO_2_ on plant growth and metabolism, here we further quantified levels of sugars, amino acids, and fatty acids in Arabidopsis exposed to a combination of periods of elevated heat and drought stress at ambient and elevated CO_2_. Such knowledge is essential for understanding plant stress responses under complex climate change scenarios.

## Materials and methods

### Plant material and growth conditions

**Fig. 1.**
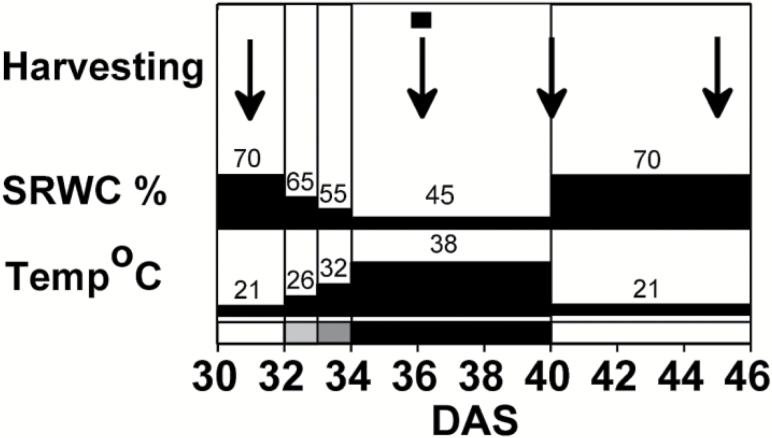
Schematic representation of the exposure regime of *Arabidopsis thaliana* (Col-0) to extremes of climate (elevated heat combined with drought, HD). At 32 d after sowing (DAS) the temperature was increased gradually and water was withheld. The grey-scale on the bottom axis indicates the step-wise temperature increase: light grey, 26/22 ºC (day/night) on 32 DAS; dark grey, 32/26 ºC on 33 DAS; black, 38/30 ºC on 34 DAS. After decreasing to 45%, soil RWC was kept constant. Recovery started at 40 DAS. Sampling time-points for metabolite analyses are indicated by the arrows, and the microarray analysis is indicated by the square (at 36 DAS).

*Arabidopsis thaliana* L. (Columbia) seeds were sown and stratified in potting mix (Tref EGO substrates, Moerdijk, The Netherlands; 5 × 5-cm pots), and grown in walk-in climate chambers (Weiss Technik, Liedekerke, Belgium) at ambient (380 ppm, two chambers) or elevated CO_2_ (730 ppm, two chambers), supplied with 150 µmol PAR m^–2^ s^–1^, 16/8 h day/night photoperiod, 21/18 °C air temperature, and 60/70% humidity (Zinta *et al.*, 2014). One-week-old seedlings were thinned to one plant per pot. To avoid pseudo-replication and in-chamber heterogeneity, the positions of pots within a chamber were rotated on a daily basis, and pots were switched between the two chambers within a treatment every 2 d from the start of the experiment. Soil relative water content (RWC) was adjusted by weighing the pots daily and watering them to 70% RWC, the optimal water requirement. At 32 d after sowing (DAS) plants were subjected to the experimental extreme climate conditions by imposing a combination of heat and drought treatments (see Fig. 1). The temperature was increased step-wise to 26/22 °C (day/night) on 32 DAS (light-grey bar at the base of Fig. 1); to 32/26 °C on 33 DAS (dark-grey bar); and to 38/30 °C at 34 DAS (black bar), and kept at this level until 40 DAS. Water was withheld from 32 DAS until the soil RWC reached 45%, which was maintained until 40 DAS. After the stress, plants were re-watered to 70% RWC and the temperature was reset to 21/18 °C. The four experimental treatments were: (i) ambient CO_2_ (labelled ‘C’); (ii) elevated CO_2_ (‘CO_2_’); (iii) heat and drought under ambient CO_2_ (‘HD’); and (iv) heat and drought under elevated CO_2_ (‘HD+CO_2_’). Whole-plant rosettes (without inflorescences) were harvested between 10:00–12:00 (the photoperiod started at 07:00), at 32 DAS (i.e. before stress exposure), at 4 d and 8 d of stress exposure (36 DAS and 40 DAS, respectively), and after recovery (45 DAS). For each treatment, five independent samples of 12 rosettes were collected, making a sample size of 60 individual plants. Harvested samples were immediately frozen in liquid nitrogen, and stored at –80 °C before analysis.

### Metabolite and enzyme determinations

To examine changes in primary metabolism at the biochemical level, cellular concentrations of sugars, amino acids, and fatty acids were determined. Soluble sugar concentrations were determined using high-performance anion-exchange chromatography with pulsed amperometric detection (HPAEC-PAD; Dionex, Sunnyvale, CA, USA) (Vergauwen *et al.*, 2000). Briefly, 100 mg (FW) plant material was ground in liquid nitrogen using a MagNA Lyser (Roche, Vilvoorde, Belgium). One ml of 50 mM TAE extraction buffer (0.02% sodium azide, 10 mM mannitol, 0.1% polyclar, 10 mM NaHSO_3_, 1 mM mercapto-ethanol, 1 mM phenylmethanesulfonylfluoride, pH 7.5) was added and the mixture was further homogenized with the MagNA Lyser. The extract was centrifuged and heated, and glucose, fructose, sucrose, and raffinose were determined after separation on a mixed bed Dowex ion exchange column (Acros Organics, Morris Plains, NJ, USA) (Vergauwen *et al.*, 2000; AbdElgawad *et al.*, 2014). Total soluble sugar was calculated as the sum of the measured individual soluble sugars. Starch content in the pellet remaining after soluble sugar extraction was determined enzymatically (Galtier *et al.*, 1995).

The α-amylase activity was determined in extracts of 100 mg (FW) frozen leaf material homogenized using a MagNA Lyser in 1 ml of 50 mM cold phosphate buffer (pH 5.2). After centrifugation (14 000 *g*, 4 °C, 5 min), the supernatant was used for assaying α-amylase, and an aliquot of the extract was heated (70 °C for 5 min with 3 mmol l^–1^ CaCl_2_) to inactivate β-amylase (Tárrago and Nicolás, 1976). Using a 0.2 % (w/v) boiled starch solution and 0.05 % (w/v) I_2_/KI in 0.05 % (v/v) HCl (Marambe *et al.*, 1992), the α -amylase activity was assayed as the decrease in the absorbance at 620 nm. For β-amylase activity, 100 mg (FW) leaf material was homogenized in 50 mM phosphate buffer (pH 7.0, 1% PVP, 1 mM benzamidine, 20 mM cysteine) (McCleary and Codd, 1989). After centrifugation, 50 µl of the supernatant was incubated at 37 °C with 100 µl reaction mixture (36 mM sodium phosphate buffer pH 7.0, 2U alpha-glucosidase, 0.25 µmol p-nitrophenyl-maltopentaoside). The reaction was stopped after 1 h with 1% Tris. The p-nitrophenol released from the substrate was measured at 410 nm in a microplate reader (Synergy Mx, Biotek Instruments Inc., Vermont, VT, USA).

Invertase enzyme activities were determined by homogenizing (MagNA Lyser) frozen leaf tissue in ice-cold TAE extraction buffer (pH 7.5) as described by AbdElgawad *et al.* (2014). After centrifugation (14 000 *g*, 4 °C, 15 min), the supernatant and pellet extracts were processed separately. Pellets were re-dissolved in ice-cold 50 mM Na-acetate buffer (pH 5.0) and an aliquot was subsequently used to determine cell wall invertase activity. The supernatant samples were split into two parts and precipitated with 80% saturated (NH_4_)_2_SO_4_ (incubation on ice for 30 min). After centrifugation, the pellet obtained from one part was re-dissolved in 80% (NH_4_)_2_SO_4_-saturated in TAE buffer (pH 8.5), and the pellet from the second part was re-dissolved in Na-acetate buffer (pH 5.0); aliquots were used to determine neutral and soluble acid invertase activities, respectively. Aliquots obtained for neutral, soluble, and cell wall invertase extracts were incubated (at 30 °C) with 100 μl reaction mixture containing 100 mM sucrose in TAE buffer pH 8.5 (neutral invertase) or Na-acetate buffer pH 5.0 (cell wall and soluble acid invertases), and 0.02% (w/v) Na-azide. Reactions were stopped by keeping an aliquot for 5 min in a water bath at 90 °C. The formation of fructose as a product of sucrose degradation was determined in the reaction mixture using HPAEC-PAD (Dionex, Sunnyvale, CA, USA). Protein concentrations were determined by the method of Sedmak and Grossberg (1977).

Amino acids were determined after extraction (100 mg FW, MagNA Lyser) in 1 ml 80% (v/v) ethanol, spiked with norvaline as an internal control (Sinha *et al.*, 2013). Quantitative determination was performed using a Waters Acquity UPLC-tqd chromatography system (Milford, Massachusetts, USA), equipped with an ethylene-bridged hybrid (BEH) amide 2.1 × 50 column. Total amino acid content was calculated as the sum of all individual amino acids.

For lipid profiling (Torras-Claveria *et al.*, 2014), plant samples (300 mg FW), were extracted in 10 ml methanol at room temperature until discoloration of the tissues using a MagNA Lyser. Codeine and nonadecanoic acids were added as internal standards. GC/MS analysis was carried out on a Hewlett-Packard 6890, MSD 5975 mass spectrometer (Hewlett Packard, Palo Alto, CA, USA), with a HP-5 MS column (30 m × 0.25 mm × 0.25 mm). Lipids were identified using the NIST 05 database and plant-specific databases (e.g. Golm Metabolome Database, http://gmd.mpimp-golm.mpg.de/). Total lipid content (saturated fatty acids, unsaturated fatty acids) was calculated as the sum of the individual lipids (e.g. saturated fatty acids, SFA = ∑12:0 + 13:0 + 14:0 + 15:0 + 18:0+....+26:0). The double-bond index was calculated as DBI = ∑ mol % of unsaturated fatty acids × number of double-bonds of each unsaturated fatty acid (Pamplona *et al.*, 1998).

Concentrations of metabolites are commonly expressed on a tissue fresh weight (FW), or dry weight (DW) basis, and both approaches can be justified. Our statistical analyses of the data expressed on FW or DW basis resulted in identical conclusions (data not shown); however, we prefer to present the results on a FW basis to more accurately reflect the actual cytoplasmic concentration changes impacting cell metabolism. Note that a progressive decrease in biomass during stress exposure could potentially result in ‘artificially’ elevated values, unrelated to primary metabolism changes; however, our previous data (Zinta *et al.*, 2014) indicated that there were no significant changes in biomass over the stress exposure times that we used. Moreover, many metabolite levels only transiently increased, or even decreased, over time, which is hard to explain on the basis of a progressively decreasing biomass. We are therefore confident that the metabolite changes reported here represent changes in metabolism.

### Transcriptome analysis

Transcriptome analysis was carried out on tissue samples from plants at 36 DAS using Agilent Arabidopsis (V4) 4 × 44 K arrays (Zinta *et al.*, 2014). Microarray data have been deposited at NCBI’s Gene Expression Omnibus (GEO, accession GSE57035, http://www.ncbi.nlm.nih.gov/geo/query/acc.cgi?acc=GSE57035). Genes with known functions that were significantly up- or down-regulated were organized into pathways using MapMan (Thimm *et al.*, 2004).

### Statistical analyses

The effects of high CO_2_, periods of combined elevated heat and drought, exposure time, and their interactions, were assessed by three-way ANOVA using SPSS 16.0 (SPSS Science, Woking, UK). Significant differences between means of treatments were identified using Duncan’s test (*P*<0.05). To classify metabolites into groups according to their stress response, hierarchical clustering was performed and visualized as heat maps generated with MultiExperiment Viewer (MeV) (TM4 software, Dana-Farber Cancer Institute, Boston, USA), using the Euclidian distance metric. Principal component analysis (PCA) was performed (OriginLab 9 software, OriginLab, Northampton, MA, USA) and projection on the two components with the highest explanatory values was used to make plots.

## Results

### Carbohydrate metabolism

**Fig. 2.**
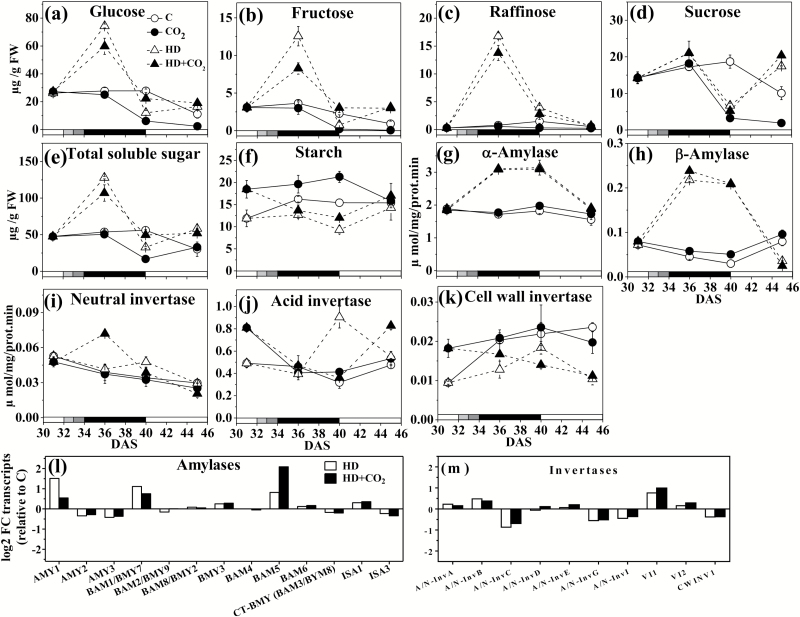
Sugar concentrations in *Arabidopsis thaliana* (Col-0) exposed to a combination of elevated heat and drought at ambient and elevated atmospheric CO_2_. (a) Glucose, (b) fructose, (c) raffinose, (d) sucrose, (e) total soluble sugars, (f) starch, (g) α-amylase activity, (h) β-amylase activity, (i) neutral invertase activity, (j) acid invertase activity, and (k) cell wall invertases activity. (l, m) Microarray-derived transcript levels at 36 d after sowing (DAS) of (l) amylases (*AMY*, α-amylase; *BAM*, β-amylase; *ISA*, iso-amylase) and (m) invertases (*A/N-Inv*, acid/neutral; *VI*, vacuolar; *CWINV*, cell wall). Data are means (±SE) (*n*=5). Treatments: C, ambient CO_2_ (control); CO_2_, elevated CO_2_; HD, combined heat and drought at ambient CO_2_; HD+CO_2_, combined heat and drought at elevated CO_2_. The shading on the bottom axes corresponds to the treatments as shown in Fig. 1.

Saccharides are important in primary carbon metabolism, and their levels and those of the enzymes controlling them were determined. Glucose, fructose, and raffinose did not change significantly between 31 and 45 DAS under non-stress conditions (Fig. 2a–c; for results of ANOVA see Supplementary Table S1 at *JXB* online). Notably, for most sugars, the heat and drought treatment caused a strong, significant (2- to 5-fold) transient increase in concentration. For glucose, fructose, and raffinose this increase occurred early after the onset of exposure to stress (36 DAS). In case, the increase was less under elevated CO_2_. The similarity in the responses of glucose, fructose, raffinose, and the total soluble sugar content (Fig. 2e) was also reflected in the cluster analysis, in which these molecules were grouped closely together (in cluster 2, see below).

Sucrose and starch showed different profiles. The sucrose concentration showed a strong decline after 36 DAS under elevated CO_2_ and stress, but relatively little change in ambient non-stressed conditions (Fig. 2d). Cellular sucrose levels are partially determined by the activities of invertase (EC 3.2.1.26). The activities of neutral and acid invertases decreased over time (*P*<0.05) in the absence of stress at both CO_2_ levels (Fig. 2i, j); however, they increased under stress at specific time points in ambient or elevated CO_2_. Cell wall (CW) invertase activity decreased under stress conditions (Fig. 2k). At the transcript level, decreased CW invertase activity corresponds to reduced expression levels of CW invertase 1 (*CWINV1*, at3g13790). For the acid and neutral invertases, increases and decreases were observed in the expression of isoform genes (Fig. 2m).

Starch content was significantly higher (*P*<0.05) under elevated CO_2_ (31–40 DAS) in non-stressed conditions (Fig. 2f). It decreased in response to heat and drought exposure, and recovered within 5 d after removing stress. The activities of α-amylase (EC 3.2.1.1) and β-amylase (EC 3.2.1.2), which are involved in determining starch levels, changed little (31–45 DAS) in ambient or elevated CO_2_ (Fig. 2g, h); however, their activities were strongly increased in the stressed plants both under ambient and elevated CO_2_. The transcripts of the amylase isoforms *AMY1* (at4g25000), *BAM1/BMY7* (at3g23920), *BAM5* (at4g15210), and *ISA1* (at2g39930) increased, whereas other amylases were somewhat less expressed under stress [e.g. *AMY2* (at1g76130), *AMY3* (at1g69830), *ISA3* (at4g09020), Fig. 2l]. Elevated CO_2_ generally reduced the impact of stress on expression, except for *BAM5* (at4g15210) where expression was further increased.

Additional results regarding stress-induced changes in carbohydrate metabolism, i.e. for sucrose–starch metabolism, glycolysis, and raffinose synthesis, were obtained from our transcriptome data at 36 DAS (ontology as defined in MapMan: see Supplementary Fig. S1, ‘Minor CHO’;) (Zinta *et al.*, 2014). Sucrose–starch metabolism heat maps showed up-regulation of amylase gene expression under stress, and a simultaneous down-regulation of starch and sucrose synthesis enzymes (sucrose-phosphate synthase, EC 2.4.1.14, at4g10120; ADP glucose pyrophosphorylase, EC 2.7.7.27, at5g19220), and sugar transporters (sucrose-proton symporter 1/plastidic GLC translocator, at5g16150; glucose-6-phosphate translocator, at5g46110). Elevated CO_2_ dampened this effect (see Supplementary Fig. S1a). Glycolysis-related genes were generally down-regulated under stress (Fig. S1b). Transcripts of raffinose synthesis genes were either up-regulated (galactinol synthase 1 and 2, at1g56600 and at2g47180; myoinositol monophosphatase-like 1, at1g31190) or down-regulated (raffinose synthases, at5g20250; galactinol synthase 3, at1g09350; Fig. S1c).

### Amino acid metabolism

**Fig. 3.**
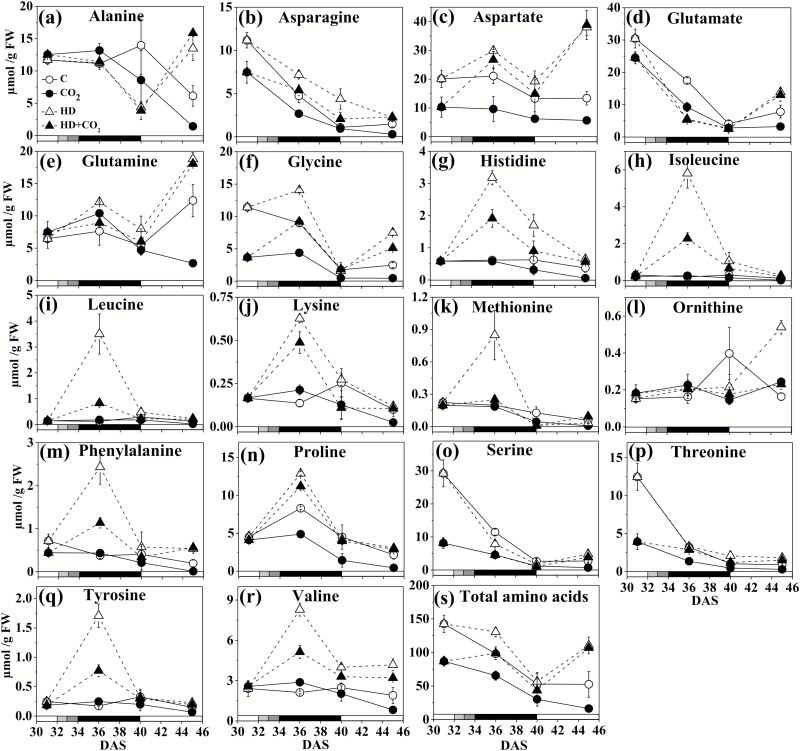
Amino acid concentrations in *Arabidopsis thaliana* (Col-0) exposed to a combination of elevated heat and drought at ambient and elevated CO_2_. (a) Alanine, (b) asparagine, (c) aspartate, (d) glutamate, (e) glutamine, (f) glycine, (g) histidine, (h) isoleucine, (i) leucine, (j) lysine, (k) methionine, (l) ornithine, (m) phenylalanine, (n) proline, (o) serine, (p) theronine, (q) tyrosine, (r) valine, and (s) total amino acids. DAS, days after sowing. Data are means (±SE) (*n*=5). Treatments: C, ambient CO_2_, i.e. control; CO_2_, control plus elevated CO_2_; HD, combined heat and drought at ambient CO_2_; HD + CO_2_, combined heat and drought at elevated CO_2_. The shading on the bottom axes corresponds to the treatments as shown in Fig. 1.

Distinct profiles of inductions and decreases were observed in the concentrations of 18 amino acids over time (Fig. 3a–r). These profiles were also identified as separate clusters in a hierarchical analysis (see below). A first group included amino acids that decreased in non-stressed and stressed plants (31–40 DAS) at both ambient and elevated CO_2_, without recovery (Asn, Glu, Ser, Thr; cluster 1 in the hierarchical analysis). In contrast, a second group showed little or no change in the absence of stress whereas a strong transient increase under heat and drought at 36 DAS was observed, which returned to pre-stress levels at 40 DAS (Gly, His, Ile, Leu, Lys, Met, Phe, Pro, Tyr, Val; cluster 2). This effect was generally dampened under elevated CO_2_. The total amino acid concentration decreased with progressing development, both in the absence of stress and under stress (Fig. 3s).

The transcriptome data at 36 DAS (Supplementary Fig. S2) showed that transcripts of pyrroline-5-carboxylate synthase (EC 1.5.1.12, at2g39800) and pyrroline-5-carboxylate reductase (EC 1.5.1.2, at5g14800) increased considerably (Supplementary Fig. S2b)_._ Expression of proline dehydrogenase decreased. These expression changes were consistent with increases in Pro. On the other hand, a putative threonine synthase transcript (EC 4.2.3.1, at1g72810), a pyridoxal-5′-phosphate-dependent enzyme Supplementary Fig. S2c), was up-regulated, but threonine levels tended to decrease. Transcripts for Met-synthesis enzymes, i.e. homocysteine methyltransferases (EC 2.1.1.10, at3g22740), SAM-dependent methyltransferase (at3g60910), and methionine adenosyltransferase (EC 2.5.1.6, at2g36880), were both up and down-regulated (Supplementary Fig. S2c). With respect to N-metabolism, transcripts of nitrate transporter (at1g12110), ammonium transporter (at2g38290), nitrate reductase 1 (EC 1.6.6.1, at1g77760), nitrite reductase (EC1.7.7.1, at2g15620), glutamine synthetase (EC 6.3.1.2, at5g16570), glutamate synthase (EC 1.4.1.13, at1g23310), and glutamate dehydrogenase (EC 1.4.1.2, at5g07440) were significantly down-regulated, whereas nitrate reductase 2 (at1g37130) was up-regulated (Supplementary Fig. S2g).

### Fatty acid metabolism

**Fig. 4.**
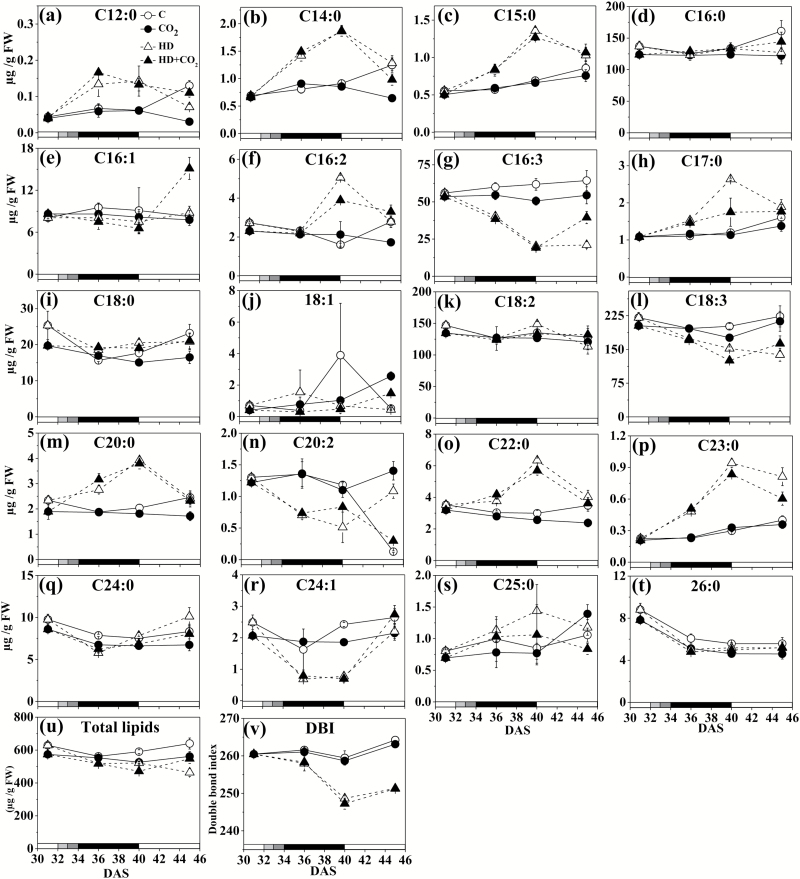
Fatty acid concentrations in *Arabidopsis thaliana* (Col-0) exposed to a combination of elevated heat and drought at ambient and elevated CO_2_. (a) Dodecanoic (C12:0), (b) tetradecanoic (C14:0), (c) pentadecanoic (C15:0), (d) hexadecanoic (C16:0), (e) hexadecenoic (C16:1), (f) hexadecadienoic (C16:2), (g) hexadecatrienoic (C16:3), (h) heptadecanoic (C17:0), (i) octadecanoic (C18:0), (j) octadecenoic (18:1), (k) octadecadienoic (C18:2), (l) octadecatrienoic (C18:3), (m) eicosanoic (C20:0), (n) eicosadienoic (C20:2), (o) docosanoic (C22:0), (p) tricosanoic (C23:0), (q) tetracosanoic (C24:0), (r) tetracosenoic (C24:1), (s) pentacosanoic (C25:0), (t) hexacosanoic (26:0), (u) total lipids, and (v) double-bond index (DBI). Data are means (±SE) (*n*=5). Treatments: C, ambient CO_2_, i.e. control; CO_2_, control plus elevated CO_2_; HD, combined heat and drought at ambient CO_2_; HD + CO_2_, combined heat and drought at elevated CO_2_. The shading on the bottom axes corresponds to the treatments as shown in Fig. 1.

Like amino acids, saturated (SFA) and unsaturated fatty acids (UFA) showed particular temporal patterns (Fig. 4a–v), and hierarchical analysis classified them into two broad groups (see below). In one group, concentrations of mostly SFAs (C12:0, C14:0, C15:0, C16:2, C17:0, C20:0, C22:0, C23:0, C25:0) increased during exposure to stress, and recovered upon re-watering (cluster 3 in the hierarchical analysis). This cluster also contain C16:0, C18:0, and C18:2, but their increases under stress was less pronounced. A second group mostly contained mono- and poly-UFAs (C16:3, C18:1, C18:3, C20:2, C24:1; cluster 4) whose concentrations decreased or remained unchanged during stress. The degree of saturation, expressed as the double-bond index (DBI), decreased significantly (*P*<0.05) during the heat and drought stress (Fig. 4v). The total lipid concentration varied little in control plants at ambient and elevated CO_2_, and decreased somewhat under stress (Fig. 4u).

Gene expression related to fatty acid chain length and saturation (MapMan bins, see Supplementary Fig S3 a, b) showed decreases in transcripts of fatty acid desaturases involved in Δ9, Δ12, and Δ15 desaturation [i.e. delta 9 desaturase 2 (at2g31360), fatty acid desaturase family protein (at1g06360), fatty acid desaturases 2, 3, 5, 7 and 8 (at3g12120, at2g29980, at3g15850, at3g11170, and at5g05580), and delta 8 sphingolipid desaturase (at3g61580)]. Decreased desaturase activity was consistent with a lower DBI (Fig. 4v). A considerable number of transcripts related to fatty acid chain length decreased in the stress treatment (e.g. acetyl-CoA carboxylase, at5g16390, EC: 6.4.1.21) (Supplementary Fig. S3a). These changes were less pronounced under elevated CO_2_. Analysis of the distribution of chain lengths showed that the proportion of fatty acids with short chains (C12, C14, C15) increased more under stress (36 and 40 DAS), whereas C16 and C18 fatty acids increased less under stress conditions (Supplementary Fig. S4).

### Hierarchical clustering and principal component analysis

**Fig. 5.**
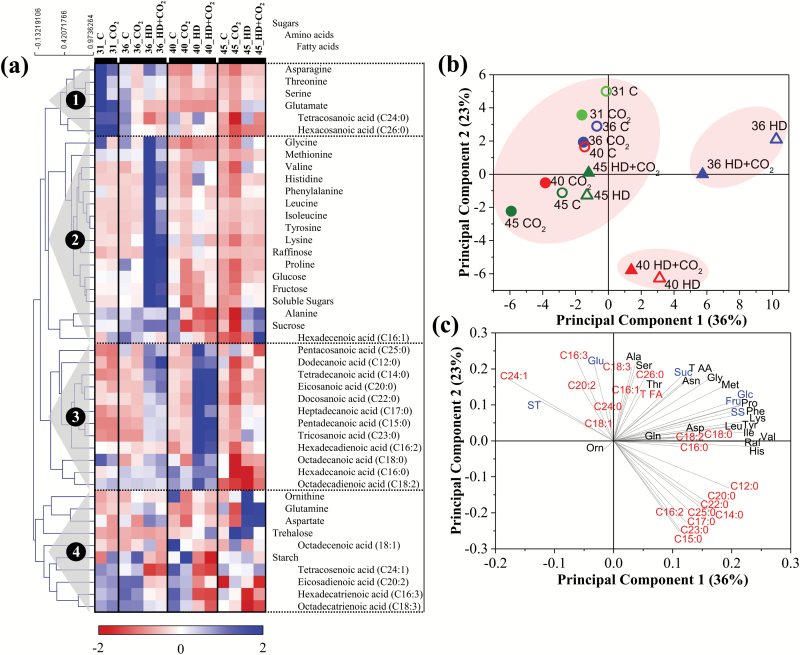
Hierarchical clustering and principal component analysis (PCA) of primary metabolite concentrations in *Arabidopsis thaliana* (Col-0) in response to a combination of elevated heat and drought under ambient and elevated CO_2_. (a) Heat map and cluster tree representation of the normalized metabolite levels, and PCA plots separating (b) the sampling time-points (c) and the measured metabolites. Treatments: C, ambient CO_2_, i.e. control; CO_2_, control plus elevated CO_2_; HD, combined heat and drought at ambient CO_2_; HD + CO_2_, combined heat and drought at elevated CO_2_.

Hierarchical clustering analysis of all the metabolite data resulted in the separation of four groups (Fig. 5a). As described above, these groups coincided well with particular patterns of time-dependent changes in metabolite concentrations. The cluster analysis also clearly illustrated the separation in response patterns between amino acids and fatty acids. To independently test the deductions of the hierarchical cluster analysis, metabolite data were subjected to principal component analysis (PCA) (Fig. 5b). The PCA plot based on the tissue sampling time-points showed a clear separation of the stress treatments from the non-stressed and recovered plants along the first two principal components (PC1 and PC2), which together explained 59% of the variability (Fig. 5b). PC1 primarily separated the treatments, whereas PC2 appeared to relate primarily to the age of the plants. PC1 (36% of the variance) was heavily determined by SFAs (C12:0, C14:0, C15:0, C17:0, C18:0, C20:0, C22:0, C23:0, C25:0), whereas PC2 (23% of the variance) showed high loading for sugars, amino acids, and poly-UFAs (Fig. 5c).

## Discussion

**Fig. 6.**
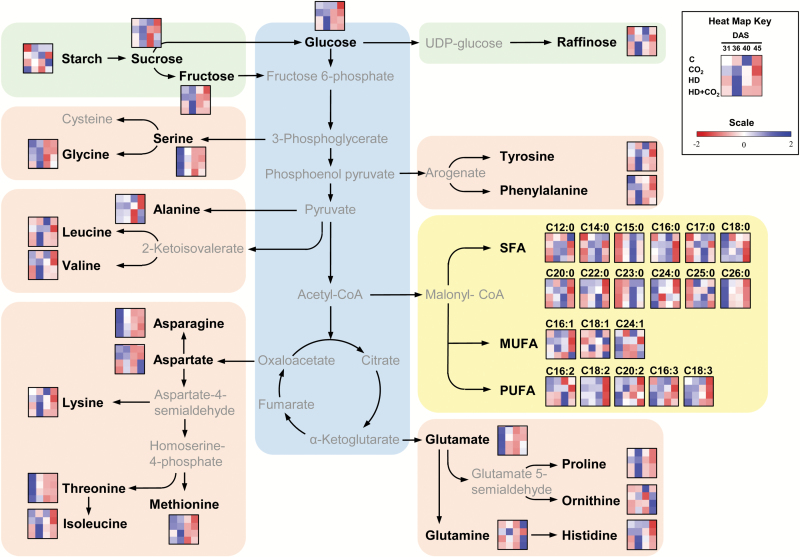
Overview of all metabolite concentrations, organised by class of molecule and biosynthetic origin. The 4 × 4 heat maps represent normalized metabolite levels at different time-points [horizontal: 31, 36, 40, 45 d after sowing (DAS)] and treatments (vertical: C, ambient CO_2_, i.e. control; CO_2_, control plus elevated CO_2_; HD, combined heat and drought at ambient CO_2_; HD + CO_2_, combined heat and drought at elevated CO_2_).

Understanding plant responses to predicted future climate stress conditions requires in-depth analysis. While many studies have focused on oxidative stress and related defence responses, much less attention has been given to effects on primary metabolism. In this study we quantified sugars, amino acids, and lipids in Arabidopsis plants exposed to climate extremes (a period of elevated heat combined with drought) at ambient and elevated CO_2_. In addition, as metabolic responses are dynamic, we sampled rosette leaves after short (4-d) and long (8-d) term stress exposure, and recovery. An overview of all metabolite changes, organised by class of molecule and biosynthetic origin is presented in Fig. 6. The results provide insights into metabolite-type and time-specific responses, and lead to new conclusions that complement our previous work (Zinta *et al.*, 2014).

### Short-term stress responses are dominated by changes in sugars and amino acids

Elevated CO_2_ alone is often observed to increase starch concentrations of plant tissues (Teng *et al.*, 2006; Ainsworth and Rogers, 2007; Ekman *et al.*, 2007; Ainsworth, 2008). We also observed that plants grown under high CO_2_ had increased starch content. Plants exposed to heat and drought stress showed lower starch levels, which was consistent with increased amylase activities, and up-regulation of α- and β-amylase transcripts. In addition, elevated ADP-glucose pyrophosphorylase (AGPase), which catalyses the first committed reaction of starch synthesis, could also have contributed to lower starch levels. Increases in amylase transcripts under heat stress have also been observed previously (Rizhsky *et al.*, 2004).

Short-term exposure to stress resulted in strong, transient increases in soluble sugars and various amino acids. Transient increases in sugars and amino acids have been observed in Arabidopsis exposed to high irradiance and sulphur depletion (Wulff-Zottele *et al.*, 2010), and to different light intensities (Jänkänpää *et al.*, 2012). This raises the question of the mechanisms that underlie metabolite changes during the stress period. It seems plausible that transient increases in the levels of sugars and amino acids are the result of decreased plant growth rate and reduced photosynthesis causing a reduction in the demand for primary metabolites for biomass. Increased levels of soluble sugars have also been observed in other species under exposure to stress (Krasensky and Jonak 2012; Hossain *et al.*, 2017), suggesting that this is a more common response in plants. It is notably that our previous analysis showed a decrease in photosynthetic activity in stressed plants (Zinta *et al*., 2014), indicating that the elevated sugars were not the result of extra C-fixation, but instead came from starch breakdown.

For some amino acids, the increased level was supported by altered transcript levels of key biosynthesis genes. For example, transcripts of pyrroline-5-carboxylate synthase and pyrroline-5-carboxylate reductase increased under stress conditions. Both enzymes function as positive regulators of Pro biosynthesis (AbdElgawad *et al.*, 2015). In addition, transcripts for proline dehydrogenase decreased, consistent with the increase in Pro, and the changes in transcript levels for all these genes were suppressed under elevated CO_2_. On the other hand, however, the expression of methionine-synthesis enzymes was both up- and down-regulated, and therefore did not clearly explain the increased methionine levels. The expression of putative threonine synthase was up-regulated, while Thr levels tended to decrease. These observations illustrate the limitations in using gene transcription analysis to explain metabolite-level changes, and extensive enzyme activity measurements are necessary to understand the discrepancies. It should be noted that the plant material harvested for transcript and metabolite analyses was not homogeneous, as it contained old and young leaves, and mature and dividing tissues, and responses may vary at different developmental stages (Beemster *et al.*, 2005; Avramova *et al.*, 2015, 2017). Nevertheless, the full rosette of Arabidopsis mostly contains mature leaves and hence the data most likely reflect metabolic changes in fully differentiated cells. It was notable that transiently elevated amino acids occurred in each of the main amino acid biosynthesis branches (Fig. 6) (Buchanan *et al.*, 2015); however, not all amino acids in each branch followed this transient induction pattern.

It is pertinent to try to understand the physiological importance of the transient accumulation of these particular sugars and amino acids. Their elevated levels may possibly be related to stress defence. For example, raffinose can act as a chaperone as well as an osmoprotectant (Panikulangara *et al.*, 2004; Egert *et al.*, 2013), and some monosaccharides may provide protection against specific reactive oxygen species (Valluru and Van den Ende, 2008; Keunen *et al.*, 2013). As for the amino acids, 10 out of the 18 quantified molecules were in cluster 2, and included hydrophobic, polar, and positively charged examples. Changes in amino acids may be related to changes in N-metabolism (Rogers *et al.*, 2006; Gargallo‐Garriga *et al.*, 2015). Analysing transcriptome data revealed that stress resulted in the down-regulation of key genes related to nitrate uptake, translocation, and assimilation, which suggested that N-metabolism was adversely affected by stress exposure, as observed previously (Goel and Singh, 2015). On the other hand, Pro is well known as an osmolyte and possibly also has antioxidant capacity (Szabados and Savouré, 2010), and Gly and Met are involved in the synthesis of the osmolyte glycine-betaine (Kanani *et al.*, 2010). In addition, other branched-chain amino acids (e.g. Leu, Ile, Val) are known to be osmoprotectants and accumulate under stress (Obata and Fernie, 2012). As these functions of amino acids have been demonstrated in very different species, it appears that this protective role is fairly common in plants. In summary, it appears that the rapid responses to stress exposure are related to the provision of energy and the synthesis of defense molecules.

### Long-term stress responses are dominated by changes in lipids

Two main metabolite clusters contained saturated (cluster 3) and unsaturated (cluster 4) fatty acids (Fig. 5a). SFAs mostly increased under combined heat and drought stress, with the highest level at 40 DAS, whereas UFAs mostly decreased. Elevated CO_2_ had little impact on these responses. The shift in saturation was also reflected in the DBI, and was consistent with decreased transcript levels of desaturase at 36 DAS. The strong increases in SFAs and decreases in UFAs after prolonged stress may possibly be related to adaptation of membranes to control temperature-induced increases in fluidity. Consistent with this observation, it has been previously shown that short exposure of Arabidopsis to high temperature did not cause alterations in membrane lipids, whereas prolonged stress significantly modified them (Falcone *et al.*, 2004). In addition, increase in the levels of SFAs may provide protection against heat stress (Horváth *et al.*, 1998; Nishiyama *et al.*, 1999; Grover *et al.*, 2000; Larkindale and Huang, 2004; Burgos *et al.*, 2011). Decreases in poly-UFAs may constitute a strategy to reduce high temperature-induced oxidative membrane damage (Falcone *et al.*, 2004; Liu and Bingru, 2004). Moreover, stress induced an increase in the proportion of the shorter acids in the total fatty acid fraction. This was consistent with the reduced expression of genes responsible for elongating chain length. Taken together, the prolonged impact of high temperature and water deficit stress was particularly apparent in the lipids, and occurred at the level of saturation and chain elongation. Presumably, such changes in lipids counter the impact of stress.

### Elevated CO_2_ lessens the impact of climate extremes

Elevated CO_2_ affects plant metabolism at many levels (Takatani *et al.*, 2014; Noguchi *et al.*, 2015; Abadie *et al.*, 2016), and generally has a dampening effect on the impact of abiotic stress responses (Zinta *et al.*, 2014; Roy *et al.*, 2016). This effect is caused by stomatal (e.g. transpiration) and non-stomatal (e.g. antioxidants, osmolytes, photorespiration) factors (Ghannoum *et al.*, 2003; AbdElgawad *et al.*, 2016). Elevated CO_2_ suppresses photorespiration, resulting in less ROS (mostly H_2_O_2_) and lower oxidative pressure. Increases in antioxidants have also been observed in stress- and CO_2_-treated plants, and contribute to the reduced impact of stress (Erice *et al.*, 2007; Geissler *et al.*, 2010; Farfan-Vignolo and Asard, 2012; Pintó-Marijuan *et al.*, 2013; Zinta *et al.*, 2014). Together, these factors are the basis for the reduced response to stress that were observed for the sugars and amino acids. However, it should be noted that elevated CO_2_ had little or no effect on the response of fatty acids, irrespective of stage of exposure. This points to a rather specific effect of high CO_2_, which is conceivable given that changes in H_2_O_2_ are likely to result in changes in (redox) signalling. We are not aware of previously published reports that demonstrate an effect of CO_2_ that is specific to a class of molecule. It would be of considerable interest to investigate this specific metabolic CO_2_ effect in other species, for example in crop plants, and species with a C_4_-fixation pathway.

In summary, we identified temporal changes in the primary metabolism of Arabidopsis during exposure to extreme climate conditions under both ambient and elevated CO_2_. Responses to heat and water deficit varied across different stress exposure times, and the dynamics were specific to particular classes of molecules. Sugars and the majority of amino acids tended to increase transiently after shorter exposure times, whereas fatty acid levels increased more gradually. The transient increases in the sugars and amino acids may be related to the arrest of growth under stress, causing a reduction in demand for primary metabolites. Fatty acids also showed decreasing levels of saturation, possibly to control membrane fluidity. Elevated CO_2_ reduced the impact of stress and, interestingly this effect was also specific to different classes of molecules.

## Supplementary data

Supplementary data are available at *JXB* online.

Fig. S1. Expression changes of genes related to sugar metabolism in Arabidopsis exposed to a combination of elevated heat and drought at ambient and elevated CO_2_.

Fig. S2. Expression changes of genes related to amino acid metabolism in Arabidopsis exposed to a combination of elevated heat and drought at ambient and elevated CO_2_.

Fig. S3. Expression changes of genes related to lipid metabolism in Arabidopsis exposed to a combination of elevated heat and drought at ambient and elevated CO_2_.

Fig. S4. Chain length distribution of fatty acids in Arabidopsis exposed to a combination of elevated heat and drought at ambient and elevated CO_2_.

Table S1. Results of three-way ANOVA for the changes in metabolites in relation to high CO_2_, stress, and time.

Supplementary Material
