# Supplementary material for: Dynamics of metabolic responses to periods of combined heat and drought in Arabidopsis thaliana under ambient and elevated atmospheric CO2
Source: J Exp Bot. 2018 Feb 15;69(8):2159–70. doi: 10.1093/jxb/ery055 (PMC6019062; doi:10.1093/jxb/ery055)
Supplement: Supplementary Material [file ery055_suppl_supplementary_material.pdf]

Dynamics of metabolic responses to combined heat and drought spells in *Arabidopsis thaliana* under ambient and rising atmospheric CO_2

Gaurav Zinta[1,2,5*], Hamada AbdElgawad[2,3*], Darin Peshev[4], James T. Weedon[1], Wim Van den Ende[4], Ivan Nijs[1], Ivan A. Janssens[1], Gerrit T.S. Beemster[2], Han Asard[2]

Legends of the Supplementary Data

Figure S1: Expression changes of genes related to sugar metabolism, in *Arabidopsis thaliana* (Col-0), exposed to a combination of heat wave and drought at ambient and elevated CO_2. Changes are expressed relative to control conditions. Classification is based on MapMan.

Figure S2: Expression changes of genes related to amino acid metabolism, in *Arabidopsis thaliana* (Col-0), exposed to a combination of heat wave and drought at ambient and elevated CO_2. Changes are expressed relative to control conditions. Classification is based on MapMan.

Figure S3: Expression changes of genes related to lipid metabolism, in *Arabidopsis thaliana* (Col-0), exposed to a combination of heat wave and drought at ambient and elevated CO_2. Changes are expressed relative to control conditions. Classification is based on MapMan.

Figure S4: Distribution of fatty acids chain lengths. Treatments: ambient CO_2, i.e. control (C); control plus elevated CO_2 (CO_2); combined heat and drought at ambient CO_2 (HD); and combined heat and drought at elevated CO_2 (HD + CO_2).

Table S1: Three way ANOVA analysis of the metabolite changes. The effects of high CO_2, stress, time, and their interactions were analysed.

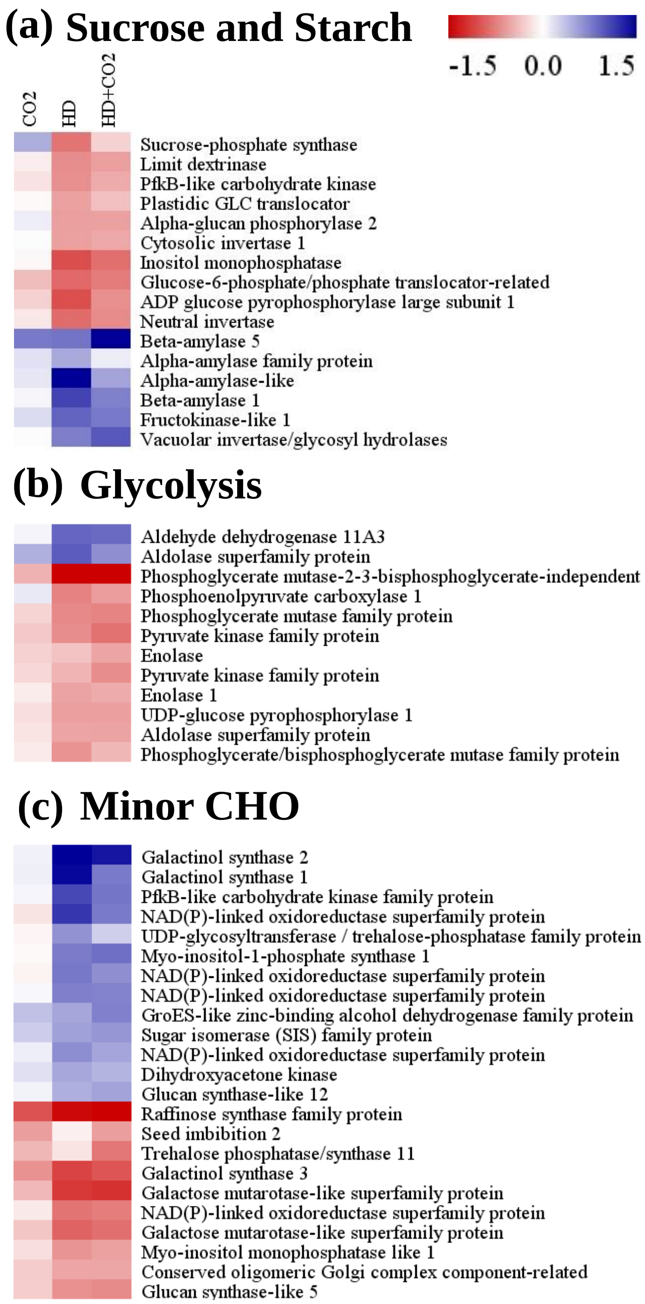

Figure S1: Expression changes of genes related to sugar metabolism, in *Arabidopsis thaliana* (Col-0), exposed to a combination of heat wave and drought at ambient and elevated CO_2. Changes are expressed relative to control conditions. Classification is based on MapMan.

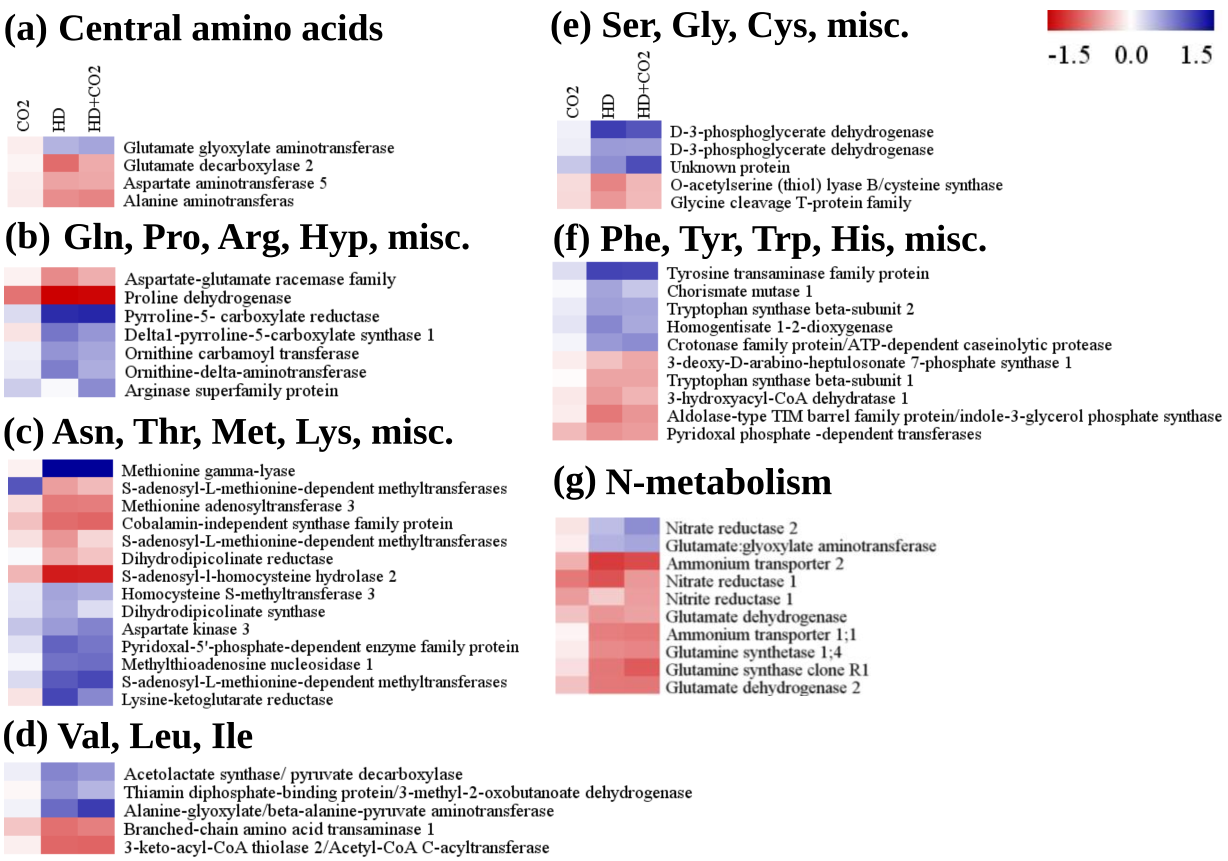

Figure S2: Expression changes of genes related to amino acid metabolism, in *Arabidopsis thaliana* (Col-0), exposed to a combination of heat wave and drought at ambient and elevated CO_2. Changes are expressed relative to control conditions. Classification is based on MapMan.

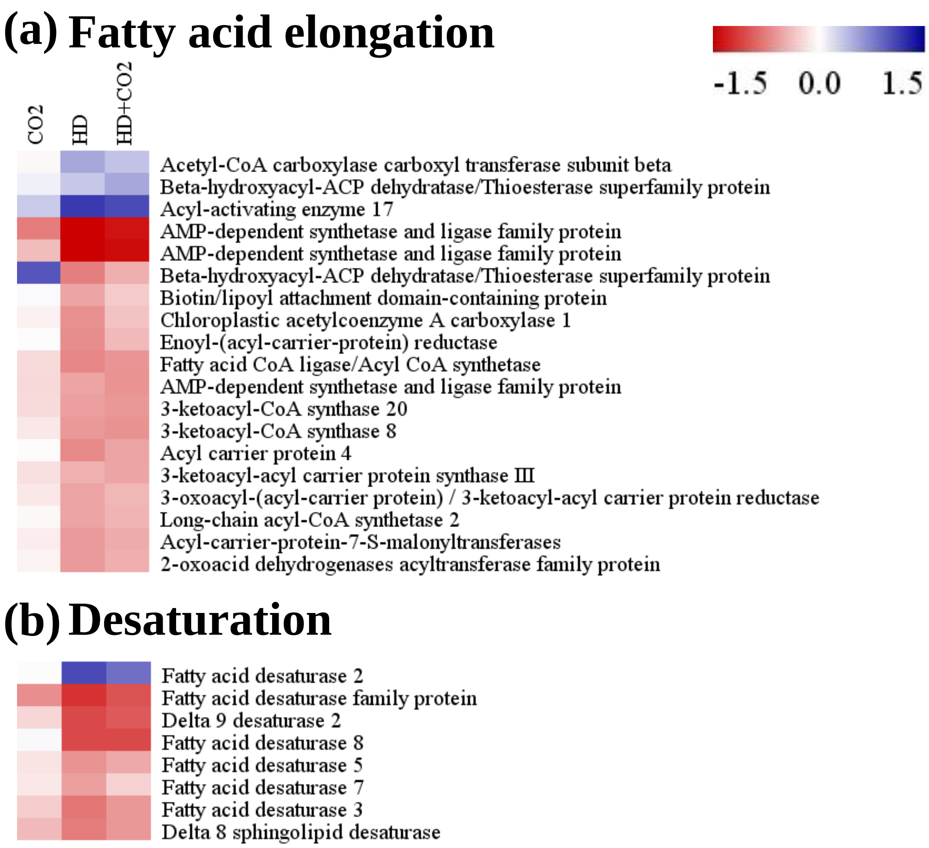

Figure S3: Expression changes of genes related to lipid metabolism, in *Arabidopsis thaliana* (Col-0), exposed to a combination of heat wave and drought at ambient and elevated CO_2. Changes are expressed relative to control conditions. Classification is based on MapMan.

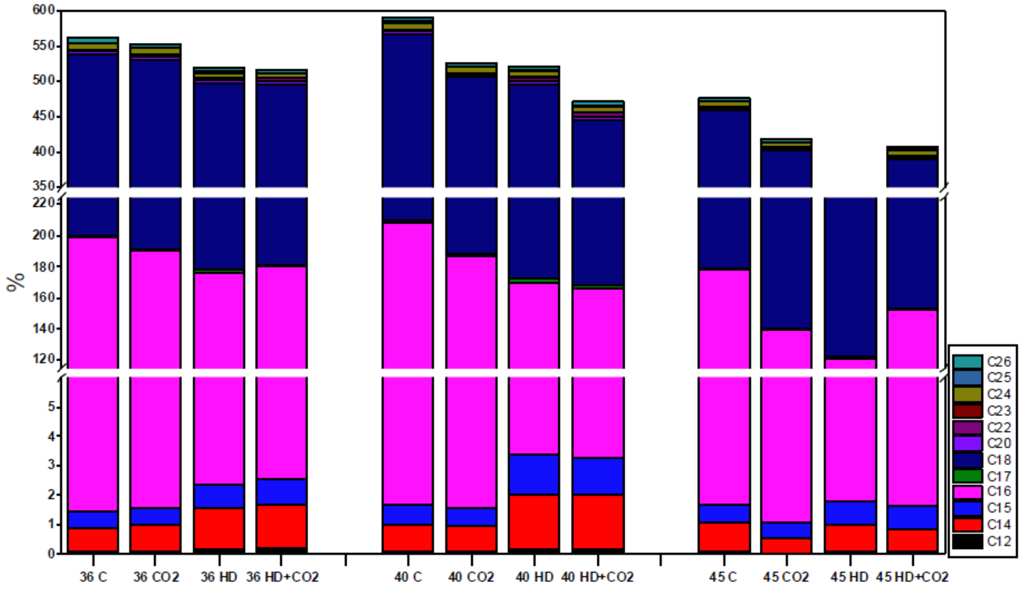

Figure S4: Distribution of fatty acids chain lengths. Treatments: ambient CO_2, i.e. control (C); control plus elevated CO_2 (CO_2); combined heat and drought at ambient CO_2 (HD); and combined heat and drought at elevated CO_2 (HD + CO_2).

Table S1: Three way ANOVA analysis of the metabolite changes. The effects of high CO_2, stress, time, and their interactions were analysed.

**Metabolite**	**CO_2**		**Stress**		**Time**		**CO_2 * Stress**		**CO_2 * Time**		**Stress * Time**		**CO_2 * Stress * Time**	
	**F value**	**P value**	**F value**	**P value**	**F value**	**P value**	**F value**	**P value**	**F value**	**P value**	**F value**	**P value**	**F value**	**P value**
**Ornithine**	6.157	**0.020**	1.844	0.187	3.049	0.066	1.144	0.295	3.307	0.054	4.959	**0.016**	8.013	**0.002**
**Lysine**	7.320	**0.012**	28.256	**<0.001**	35.805	**<0.001**	1.441	0.242	1.755	0.194	19.404	**<0.001**	2.198	0.133
**Glutamine**	7.972	**0.009**	36.332	**<0.001**	25.520	**<0.001**	0.132	0.720	3.633	**0.042**	14.788	**<0.001**	7.702	**0.003**
**Asparagine**	15.357	**0.001**	41.942	**<0.001**	44.747	**<0.001**	0.124	0.728	1.568	0.229	1.210	0.316	2.513	0.102
**Alanine**	0.701	0.411	0.662	0.424	3.882	**0.035**	1.961	0.174	0.960	0.397	19.459	**<0.001**	1.258	0.302
**Threonine**	16.239	**<0.001**	22.604	**<0.001**	28.017	**<0.001**	1.519	0.230	1.106	0.347	0.184	0.833	1.774	0.191
**Glycine**	75.232	**<0.001**	146.131	**<0.001**	254.298	**<0.001**	0.265	0.611	18.508	**<0.001**	25.190	**<0.001**	1.164	0.329
**Valine**	17.516	**<0.001**	140.729	**<0.001**	32.311	**<0.001**	9.229	**0.006**	0.705	0.504	13.864	**<0.001**	8.325	**0.002**
**Serine**	62.503	**<0.001**	0.093	0.762	103.754	**<0.001**	7.648	**0.011**	15.895	**<0.001**	16.801	**<0.001**	1.894	0.172
**Proline**	4.603	**0.042**	11.896	**0.002**	30.440	**<0.001**	1.638	0.213	0.341	0.715	2.925	0.073	0.062	0.940
**Isoleucine**	15.057	**0.001**	73.537	**<0.001**	45.648	**<0.001**	12.462	**0.002**	8.492	**0.002**	40.646	**<0.001**	10.621	**<0.001**
**Leucine**	14.042	**0.001**	30.009	**<0.001**	20.873	**<0.001**	11.406	**0.002**	8.168	**0.002**	20.624	**<0.001**	10.583	**0.001**
**Methionine**	7.674	**0.011**	6.421	**0.018**	27.151	**<0.001**	2.581	0.121	5.473	**0.011**	10.010	**0.001**	7.586	**0.003**
**Histidine**	18.671	**<0.001**	95.281	**<0.001**	38.798	**<0.001**	5.231	**0.031**	1.595	0.224	18.221	**<0.001**	3.784	**0.037**
**Phenylalanine**	7.078	**0.014**	33.306	**<0.001**	18.744	**<0.001**	3.011	0.096	2.007	0.156	10.555	**0.001**	4.521	**0.022**
**Glutamate**	15.445	**0.001**	0.215	0.647	47.727	**<0.001**	13.308	**0.001**	2.547	0.099	54.901	**<0.001**	2.780	0.082
**Aspartate**	6.439	**0.018**	57.799	**<0.001**	8.926	**0.001**	2.276	0.144	0.271	0.765	8.952	**0.001**	0.198	0.821
**Tyrosine**	11.272	**0.003**	49.748	**<0.001**	38.258	**<0.001**	6.465	**0.018**	4.801	**0.018**	33.272	**<0.001**	10.785	**<0.001**
**Glucose**	20.400	**<0.001**	179.627	**<0.001**	280.015	**<0.001**	16.502	**<0.001**	1.558	0.231	88.125	**<0.001**	24.030	**<0.001**
**Fructose**	8.980	**0.006**	116.800	**<0.001**	118.471	**<0.001**	0.537	0.471	5.990	**0.008**	36.014	**<0.001**	13.097	**<0.001**
**Sucrose**	22.146	**<0.001**	24.971	**<0.001**	72.103	**<0.001**	28.658	**<0.001**	12.094	**<0.001**	47.526	**<0.001**	9.267	**0.001**
**Raffinose**	14.961	**0.001**	496.913	**<0.001**	302.766	**<0.001**	1.710	0.203	3.238	0.057	288.049	**<0.001**	4.132	**0.029**
**Soluble Sugar**	13.514	**0.001**	185.733	**<0.001**	163.385	**<0.001**	4.502	**0.044**	2.104	0.144	61.124	**<0.001**	25.909	**<0.001**
**Starch**	0.395	0.535	23.986	**<0.001**	0.734	0.490	10.131	**0.004**	1.027	0.373	7.081	**0.004**	0.938	0.405
**C12:0**	0.446	0.511	30.960	**<0.001**	6.808	**0.005**	4.954	0.036	1.124	0.342	6.467	**0.006**	2.567	0.098
**C14:0**	6.341	**0.019**	237.839	**<0.001**	89.155	**<0.001**	1.183	0.288	11.318	**<0.001**	42.579	**<0.001**	0.990	0.386
**C15:0**	0.886	0.356	199.246	**<0.001**	59.852	**<0.001**	0.039	0.846	0.761	0.478	31.241	**<0.001**	0.937	0.406
**C16:0**	1.261	0.273	0.054	0.819	24.275	**<0.001**	8.524	**0.008**	0.671	0.520	0.582	0.566	2.468	0.106
**C17:0**	8.257	**0.008**	59.479	**<0.001**	14.806	**<0.001**	4.112	0.054	4.058	**0.030**	11.609	**<0.001**	3.688	0.040
**C18:0**	3.511	0.073	13.816	**0.001**	8.473	**0.002**	2.353	0.138	3.069	0.065	1.577	0.227	1.931	0.167
**C20:0**	1.448	0.241	178.533	**<0.001**	79.736	**<0.001**	4.342	**0.048**	3.617	**0.042**	38.145	**<0.001**	0.563	0.577
**C22:0**	11.574	**0.002**	278.725	**<0.001**	120.918	**<0.001**	2.641	0.117	4.691	**0.019**	65.686	**<0.001**	1.799	0.187
**C23:0**	7.917	**0.010**	738.287	**<0.001**	120.704	**<0.001**	9.399	**0.005**	5.769	**0.009**	66.510	**<0.001**	3.716	**0.039**
**C24:0**	30.554	**<0.001**	0.079	0.781	13.613	**<0.001**	1.391	0.250	3.553	**0.044**	19.820	**<0.001**	3.678	0.040
**C25:0**	0.878	0.358	1.295	0.266	0.776	0.472	0.679	0.418	0.237	0.791	1.597	0.223	0.429	0.656
**C26:0**	9.333	**0.005**	1.522	0.229	28.531	**<0.001**	4.799	**0.038**	0.234	0.793	1.940	0.166	0.002	0.998
**C16:1**	0.046	0.831	0.001	0.977	0.604	0.555	1.485	0.235	1.867	0.176	3.907	**0.034**	1.213	0.315
**C18:1**	0.198	0.660	1.156	0.293	0.579	0.568	0.005	0.943	1.640	0.215	1.153	0.333	1.150	0.333
**C24:1**	0.135	0.716	43.497	**<0.001**	7.732	**0.003**	2.402	0.134	1.523	0.238	13.336	**<0.001**	1.300	0.291
**C16:2**	3.077	0.092	70.609	**<0.001**	32.860	**<0.001**	0.318	0.578	0.181	0.836	38.498	**<0.001**	10.787	**<0.001**
**C18:2**	0.412	0.527	0.262	0.613	34.926	**<0.001**	0.126	0.726	1.151	0.333	0.748	0.484	0.864	0.434
**C20:2**	2.447	0.131	30.238	**<0.001**	17.931	**<0.001**	6.880	**0.015**	0.473	0.629	6.059	**0.007**	17.857	**<0.001**
**C16:3**	2.467	0.129	331.228	**<0.001**	40.256	**<0.001**	17.339	**<0.001**	3.969	0.032	16.781	**<0.001**	3.370	0.051
**C18:3**	4.533	**0.044**	138.684	**<0.001**	60.530	**<0.001**	1.168	0.291	7.250	**0.003**	6.100	**0.007**	1.870	0.176
**DBI**	1.024	0.322	230.418	**<0.001**	38.570	**<0.001**	0.131	0.721	0.238	0.790	24.279	**<0.001**	0.197	0.823
